# Cone dysfunction in *ARR3*-mutation-associated early-onset high myopia: an electrophysiological study

**DOI:** 10.1186/s13023-024-03390-9

**Published:** 2024-10-17

**Authors:** Tamás Fehér, Noémi Széll, István Nagy, Zoltán Maróti, Tibor Kalmár, Zoltán Sohajda, Mirella T. S. Barboni

**Affiliations:** 1 Institute of Biochemistry, HUN-REN Biological Research Centre, 62 Temesvari krt., Szeged, H6726 Hungary; 2 Department of Ophthalmology, University of Debrecen, 98. Nagyerdei krt, Debrecen, H4032 Hungary; 3 Seqomics Biotechnology Ltd., Mórahalom, Hungary; 4 Genetic Diagnostic Laboratory, Department of Pediatrics, Albert Szent-Györgyi Health Centre, University of Szeged, 35-37 Temesvari krt., Szeged, H6726 Hungary; 5 Kenézy Campus Department of Ophthalmology, University of Debrecen, 2-26 Bartók Béla út, Debrecen, H4031 Hungary; 6 Department of Ophthalmology, Semmelweis University, 39 Mária u, Budapest, H1085 Hungary

**Keywords:** Early onset high myopia, Retina, *ARR3*, Electroretinogram, Cone arrestin, Cone dysfunction

## Abstract

**Background:**

Myopia-26, a Mendelian form of early-onset high-myopia (eoHM) caused by mutations in the X-chromosomal *ARR3* gene and predominantly affecting females, curiously, may provide an alternative route of investigation to unveil retinal mechanisms underlying pathological eye growth. We conducted a case-control cross-sectional prospective electrophysiological study in genetically characterized Myopia-26 patients (*ARR3* heterozygous symptomatic females) compared with high myopes harboring intact *ARR3* alleles and one carrier hemizygous male.

**Results:**

Participants were 26 volunteers: 10 healthy control females (E-CTRL, mean age = 31.5 ± 8.8 years), one healthy control male, one carrier male of the mutant *ARR3* allele and 14 female eoHM patients (mean age = 27.0 ± 13.1 years) divided in two groups: seven without (M-CTRL) and seven with (MYP-26) genetic alteration in the *ARR3* gene. The clinical evaluation included complete eye screening and full-field electroretinograms (ERGs) recorded from both eyes under mydriasis. Spherical equivalent was comparable (mean=-9.55 ± 2.46 and − 10.25 ± 3.22 for M-CTRL and MYP-26, respectively) and best corrected visual acuity (BCVA) was significantly different between M-CTRL and MYP-26 (1.0 vs. 0.406 ± 0.253, respectively). E-CTRL and M-CTRL showed similar light-adapted flash and flicker ERG amplitudes; however, the prior values were reduced by ~ 35% (a- and b-waves alike), the latter by ~ 55% in the MYP-26 group (F_(2, 45)_ > 21.821, *p* < 0.00001). Dark-adapted a-wave amplitudes were slightly reduced (by ~ 20%) in all myopic patients compared to E-CTRL, irrespective of the *ARR3* genotype (E-CTRL vs. eoHM, *p* = 0.038).

**Conclusions:**

The cone dysfunction observed in Myopia-26 patients is specifically linked to the mutation of *ARR3*, and is not the consequence of eoHM, i.e. elongation of the eye. It may play a role in myopic refractive error development through a yet unconfirmed pathomechanism.

**Supplementary Information:**

The online version contains supplementary material available at 10.1186/s13023-024-03390-9.

## Background

Early-onset progressive forms of high myopia are determined strongly and primarily by genetic factors, i.e. they are often inherited in a monogenic manner with one single causative, highly penetrant gene mutation, and with minimal environmental or behavioral influence [1]. By their nature, early onset high myopias (eoHMs) do not respond substantially to well established optical or pharmaceutical interventions to control myopia [2]. This further adds to the “malignant” nature of the disease, underlining the need for effective and specific therapeutic options. Therefore, a thorough understanding of the cellular bases and underlying pathophysiological mechanisms of myopia development in this population is essential [3, 4].

*ARR3* encoding the cone arrestin has been recently claimed to be the most frequently implicated gene for non-syndromic Mendelian eoHM by both Asian and European cohorts [2, 5–7]. Despite its X chromosomal locus, heterozygous mutations of the *ARR3* gene result in eoHM limited to females, a disease referred to as Myopia-26 (OMIM: #301010) associated with potentially blinding complications [8, 9].

In this study, we evaluated possible retinal dysfunctions exclusively caused by the heterozygous or hemizygous mutations of the *ARR3* gene using electroretinography (ERG). Although retinal alterations have been largely reported in myopia in general [10–16], and age-related cone dysfunction [17] was demonstrated for *ARR3* knockout mice in a murine model, the latter could not unambiguously be evidenced in humans [5, 6].

Here, ERG results from genetically characterized Myopia-26 patients were controlled for possible myopic effects to gain clear evidence regarding the pathomechanism connecting the *ARR3* mutant genotype to the eoHM phenotype.

## Methods

### Participants

Participants were 26 volunteers (24 female and two males) undergoing genetic, ophthalmological and electrophysiological examinations. Ten female subjects were emmetropic or low myopic (SE ≥ -2.50) controls (E-CTRL group, mean age = 31.5 ± 8.8 years). The other 14 female participants displayed eoHM (SE ≤ -6.0; myopia onset at the age of 2–3 years), but otherwise healthy subjects (mean age = 27.0 ± 13.1 years): seven female subjects (mean age = 25.7 ± 11.5 years) with a verified heterozygous *ARR3* gene mutation (MYP-26 group) while seven female subjects (mean age = 28.3 ± 15.4 years) with intact *ARR3* coding regions (*ARR3* wild type genotype) served as myopic controls (M-CTRL group). One male carrier, hemizygous for the *ARR3* mutation was also included, along with a male emmetropic control. The male carrier and the members of the MYP-26 group were all members of the same family [5]. Demographic and clinical information are provided in Table 1.

**Table 1:** Patients’ genetic and clinical information

ID	ARR3 mutation	Age	SE od	SE os	BCVA OD	BCVA OS	Fundus* (META PM)	Axial length (mm) o.d./o.s.	Macular OCT
M1	-	42	-14.00	-6.00	1.0	1.0	C1	27.8 / 25.57	Normal retinal, choroidal structure
M2	-	19	-10.00	-9.00	1.0	1.0	C1	26.51 / 25.95	Normal retinal, choroidal structure
M3	-	24	-10.25	-10.25	1.0	1.0	C1	27.86 / 28.07	Normal retinal, choroidal structure
M4	-	15	-12.00	-13.00	1.0	1.0	C1	26.13 / 26.78	Normal retinal, choroidal structure
M5	-	17	-6.25	-6.00	1.0	1.0	C1	25.4 / 25.31	Normal retinal, choroidal structure
M6	-	21	-9.00	-8.00	1.0	1.0	C1	26.11 / 25.93	Normal retinal, choroidal structure
M7	-	42	-10.00	-10.00	1.0	1.0	C1	26.5 / 26.5	Normal retinal, choroidal structure
M8	+	10	-11.50	-11.00	0.4	0.6	C1	27.1 / 26.58	Normal retinal, choroidal structure
M9	+	17	-7.75	-8.50	0.6	0.6	C1	25.54 / 25.61	Normal retinal, choroidal structure
M10	+	15	-7.25	-5.50	0.8	0.9	C1	25.23 / 24.4	Normal retinal, choroidal structure
M11	+	29	-10.00	-9.25	0.25	0.25	C2	26.26 / 25.97	Photoreceptor layer: intact Choroid: moderately thinned Sclera: visible
M12	+	32	-16.00	-15.25	0.4	0.4	C2	27.31 / 27.22	Photoreceptor layer: intact Choroid: considerably thinned Sclera: visible almost in full depth
M13	+	43	-9.50	-8.50	0.1	0.15	C2	26.6 / 26.28	Photoreceptor layer: intact Choroid: considerably thinned Sclera: visible almost in full depth
M14	+	52	-14.00	-7.00	0.04	0.2	C2	27.4 / 25.32	Photoreceptor layer: intact Choroid: extremely thinned (od > os) Sclera: visible in full depth

*The META-PM fundus appearance was categorized as Category C1 = tessellated retina or C2 = diffuse chorioretinal and peripapillary atrophy [8]. Normal retinal structure and choroidal thickness (C1); moderate to extreme attenuation of the choroidal thickness in association with no disruption or loss of the photoreceptor layer (C2). M1 – M7 = M-CTRL group and M8 – M14 = MYP-26 group

All procedures were performed in accordance with the ethical guidelines of the National Scientific and Research Ethics Committee, with the declaration of Helsinki (1964) and its later amendments or comparable ethical standards. Written informed consent was obtained from all participants included in this study, approved by the National Scientific and Research Ethics Committee of the Medical Research Council of Hungary (ETT TUKEB, registration number 58542-1/2017/EKU).

### Genetic analyses: Sanger sequencing of ARR3 exons and whole exome sequencing

Genomic DNA of patients M4, M5, M6, M7 were prepared using the GeneJet Whole Blood Genomic DNA purification Mini Kit (ThermoFisher Scientific, Waltham, MA), according to kit instructions. The coding exons of gene *ARR3* were PCR-amplified in a total of 8 PCR reactions, using Q5^®^ High-Fidelity DNA polymerase, supplemented with the Q5^®^ High GC Enhancer. Elongation time was 80 s, annealing temperature was 64 ^o^C, the number of PCR cycles was 35. Primer names, and the exons amplified are listed in Table S1 of the Supplement. The obtained PCR products were purified using the Gel/PCR DNA Isolation System (Viogene, Taiwan, China), and deposited to Seqomics Biotechnology Ltd., (Mórahalom, Hungary) for Sanger sequencing, applying the primers used for PCR amplification. The obtained sequences were compared to the relevant segment of the reference human genome (GenBank Acc. No. NC_000023.11) using the multi-alignment tool of SnapGene software (www.snapgene.com). Whole exome sequencing (WES) of three family members (symptomatic females M1, M2 and M3) were performed as described earlier [5].

### Ophthalmological examinations

Patients’ own and family medical history regarding ophthalmological disorders other than eoHM as well as any systemic diseases were collected. Best corrected visual acuity (BCVA) was monocularly measured with the Snellen chart. High myopia was specified as SE ≤ − 6.0 SE in at least one of the eyes. Slit lamp biomicroscopy and fundus ophthalmoscopy under mydriasis was carried out with Topcon SL-D701 (Topcon, Tokyo, Japan). Ultra-wide field (200^◦^) fundus images (Optos^®^ California, Optos, Marlborough, MA) were taken from each eye of the patients. Fundus appearances were assigned according to the META-PM (meta analyses of pathological myopia) classification system [8]. OCT (Heidelberg Engineering, Heidelberg, Germany) was performed to identify possible photoreceptoral degeneration.

### Electrophysiological examination

Dark-adapted (DA) and light adapted (LA) full-field ERGs were recorded from both eyes of all subjects following the recently updated [18] guidelines of the International Society for Clinical Electrophysiology of Vision (ISCEV). Pupils were dilated with a drop of mydriaticum (0.5% tropicamide). The RETeval portable ERG device was the instrument used for the stimulus presentation (DA 0.01, DA 3.0, LA 3.0 and LA 30 Hz flicker) and to record the ERG signals with sensor strips skin electrodes (LKC Technologies, United States). The skin was prepared prior to the ERG recordings with an abrasive gel (Nuprep) for removing oily surface, improving electrode contact and preventing possible skin artefacts.

The full-field ERG responses are described in Figure S2A. All subjects were light adapted to the ambient illumination prior the recordings. LA ERG responses to 3.0 cd.s/m² (standard) white flash superimposed to a steady white background (30 cd/m²) were recorded 30 times with inter-stimulus time of 0.5 s. Next, 30 Hz flicker ERG responses provided by 3.0 cd.s/m² stimulation were recorded. The right eye was tested first, followed by identical steps to test the left eye. After LA measurements, a period of 10 min of dark adaptation was included before the DA ERG recordings. Two flash stimuli were presented in this order: 0.01 cd.s/m² (weak) and 3.0 cd.s/m² (standard) flash without background and with inter-stimulus times of 2 and 10 s, respectively. As in the LA recordings, the right eye was tested first and left eye afterwards using the same protocol. The contralateral eye was completely covered during the tests.

### Signal processing and statistical analysis

Amplitude and implicit times of main ERG components were obtained using time-and frequency-domain analysis. Figure S2 shows light-adapted (Figure S2B) and dark-adapted (Figure S2C) ERG components marked with numbers. The respective names of the components are shown at the upper right panel. The amplitude of the a-wave was defined as the difference in µV between the baseline and the minimum amplitude after stimulus onset. The amplitude of the b-wave was the difference in µV between a-wave trough and the peak of the b-wave. The implicit times corresponded to the intervals between the stimulus onset and the peak component. DA 3.0 responses were analyzed twice: the original traces and filtered signals to emphasize higher frequency oscillatory potentials which were afterwards analyzed in the time-domain. ERG results were expressed as means ± SD. Group differences were analyzed using one or two-way ANOVAs depending on the presence of a within-subject repeated measure (oscillatory potentials, for instance). Bonferroni post hoc test was used to correct for multiple group comparisons. p-values < 0.05 were considered statistically significant.

## Results

### Genetic findings

Genetic and clinical findings are presented in Table 1. Genetic analyses were carried out for all the 14 eoHM patients (groups M-CTRL and MYP-26) and the asymptomatic carrier male. All MYP-26 patients (M8-M14) showed heterozygosity, while the carrier male displayed hemizygosity for the nonsense mutation (c.214 C > T, p.Arg72*) within the *ARR3* gene identified and reported previously [5]. The DNAs from the eoHM patients of the M-CTRL group were verified to harbor wild type *ARR3* CDS only, using either whole exome sequencing (for patients M1-M3) or Sanger sequencing (for patients M4-M7), as described in the Methods.

### Clinical and ophthalmological findings

**Fig. 1:**
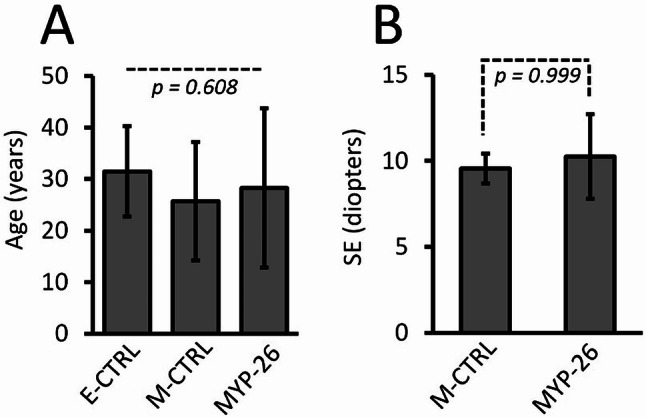
Mean (± SD) age and spherical equivalent (SE) of the investigated groups. The three groups showed comparable mean age: E-CTRL = 31.5 ± 8.5, M-CTRL = 25.7 ± 11.0 and MYP-26 = 28.3 ± 14.8 (**A**) and the two groups with eoHM showed comparable mean spherical equivalent: M-CTRL = -9.55 ± 2.46 SE and MYP-26 = -10.25 ± 3.22 (**B**)

Medical history and ophthalmological examinations revealed no systemic or ocular disorders other than eoHM in M-CTRL and MYP-26 patients, i.e. there were no clues for a syndromic cause of eoHM. Figure 1A shows that the three groups, E-CTRL, M-CTRL and MYP-26, were age matched (F_(2, 21)_ = 0.509, *p* = 0.608, Bonferroni *p* > 0.9). E-CTRL subjects were mostly emmetropes, with spherical equivalents (SE) from − 0.50 to + 0.50. All 14 M-CTRL and MYP-26 patients had eoHM (Table 1). The two myopic groups were also matched for refraction (Fig. 1B): SE_M−CTRL_= -9.55 ± 2.46; SE_MYP−26_= -10.25 ± 3.22 (Bonferroni *p* = 0.999). Best-corrected visual acuities (BCVAs) were reduced for all MYP-26 patients to various extents (from mildly affected to severely impaired; mean = 0.406 ± 0.253). All subjects in the M-CTRL group however, showed normal BCVA (1.0 for both eyes) (unpaired T-test: *p* = 5.77 × 10^− 7^).

**Fig. 2:**
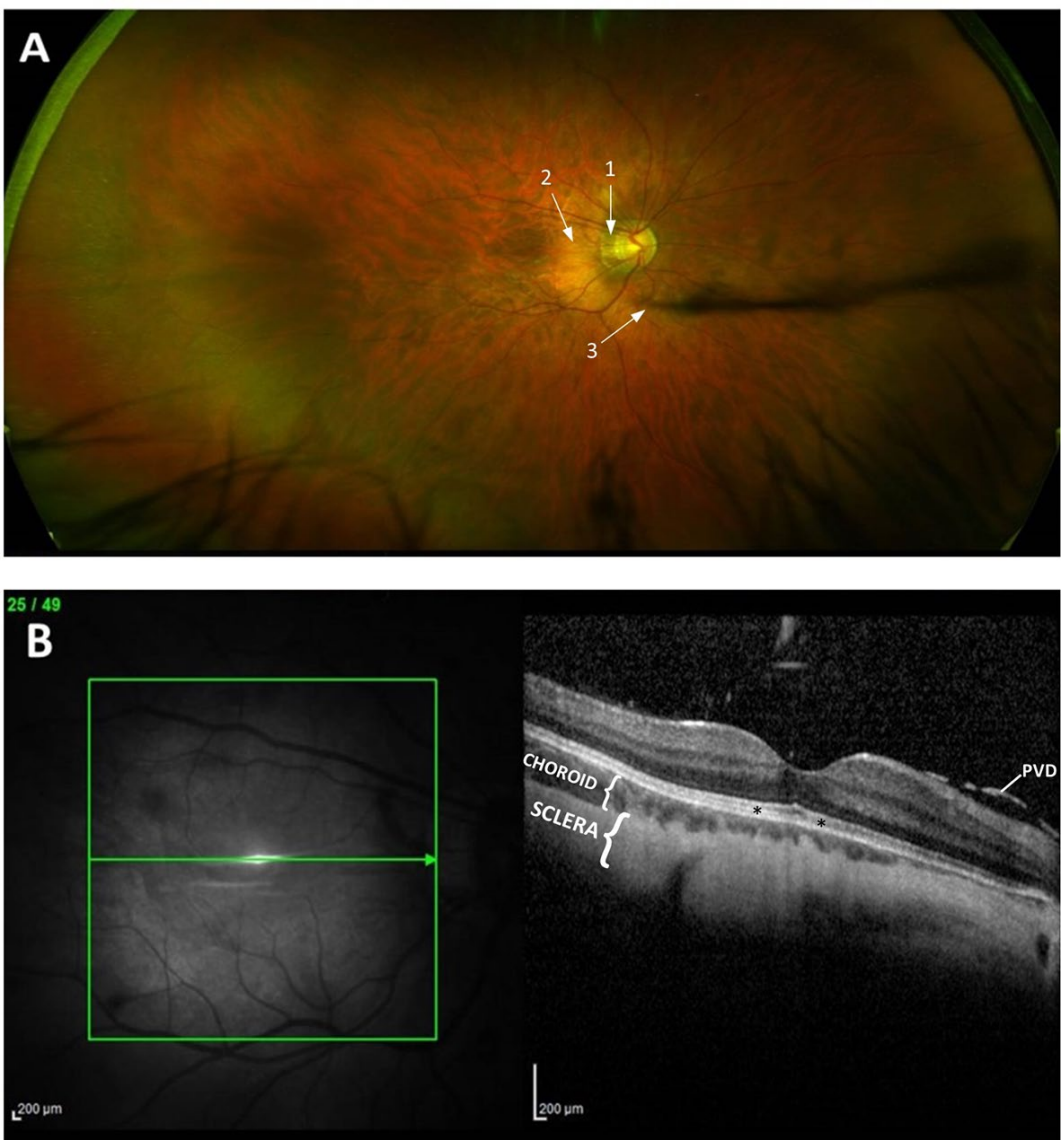
Fundus and OCT images for a representative of the MYP-26 group. (**A**) Ultra-widefield (Optos^®^ California) fundus image of the right eye displaying a META-PM C2 fundus: tessellated appearance of the retina along with peripapillary (1) and diffuse chorioretinal atrophy (2), and a posterior vitreous detachment (PVD) (3). The corresponding macular OCT image (**B**) displaying a considerably thinned choroid, and the sclera visible almost in its full depth. Photoreceptor layer was intact (asterisks) and PVD was observed

Fundus examinations and optical coherence tomography (OCT) results are described in Table 1 and a representative fundus and OCT image of a MYP-26 patient is shown in Fig. 2. No characteristics of cone dystrophy - expected based on results of the murine *ARR3* knockout model [17] were observed. Retinal morphology was rather characteristic of high myopia in both myopic groups (M-CTRL and MYP-26). The META-PM classification system was used to categorize myopic retinal changes [8]. Normal retinal structure (Category 0) was observed in the E-CTRL and in the male carrier patients. Tesselated retina (Category 1) was observed in all M-CTRL subjects, while diffuse chorioretinal atrophy (Category 2) was observed only for advanced age MYP-26 patients. Advanced stages of pathological fundus alterations (META-PM C3-4), signs of posterior staphyloma or any visual media opacities that could affect light propagation and thus electrophysiology tests, were not observed in these patients. In addition, axial lengths were consistent with the myopic level and similar in both myopic groups (Table 1).

### Electrophysiological findings

**Fig. 3:**
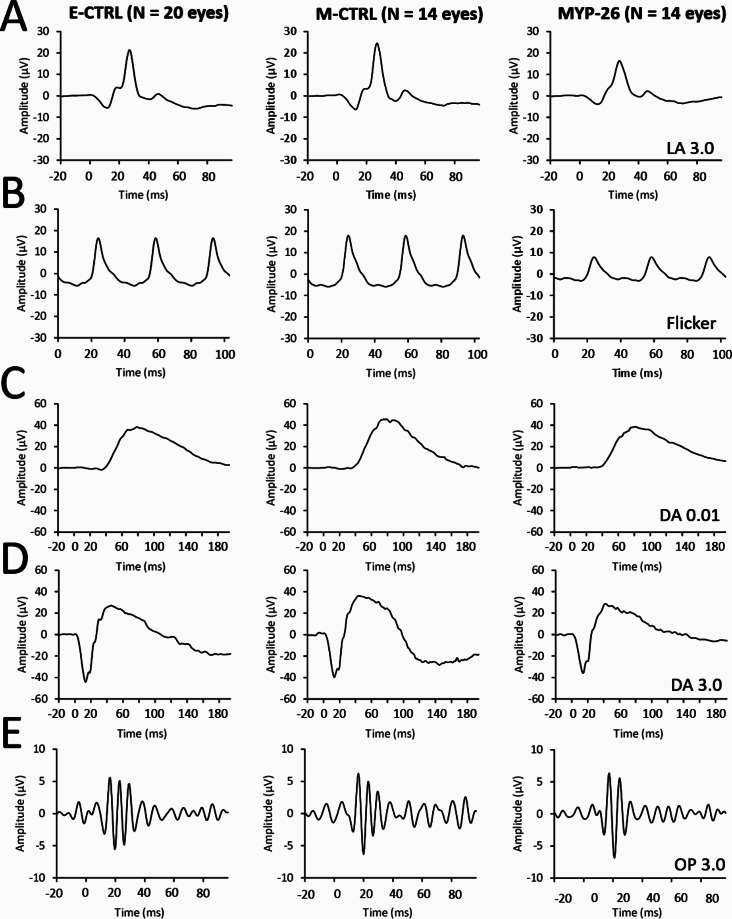
Group ERG responses: E-CTRL, M-CTRL and MYP-26. From top to bottom: light-adapted 3.0 cd.s/m² (**A**), light-adapted flicker (**B**), dark-adapted 0.01 cd.s/m² (**C**), dark-adapted 3.0 cd.s/m² (**D**) and oscillatory potentials extracted from dark-adapted 3.0 cd.s/m² signals (**E**). First column shows the averaged control traces (E-CTRL, *N* = 20 eyes), second column traces from M-CTRL group (*N* = 14 eyes) and third column shows mean traces of MYP-26 group (*N* = 14 eyes)

**Fig. 4:**
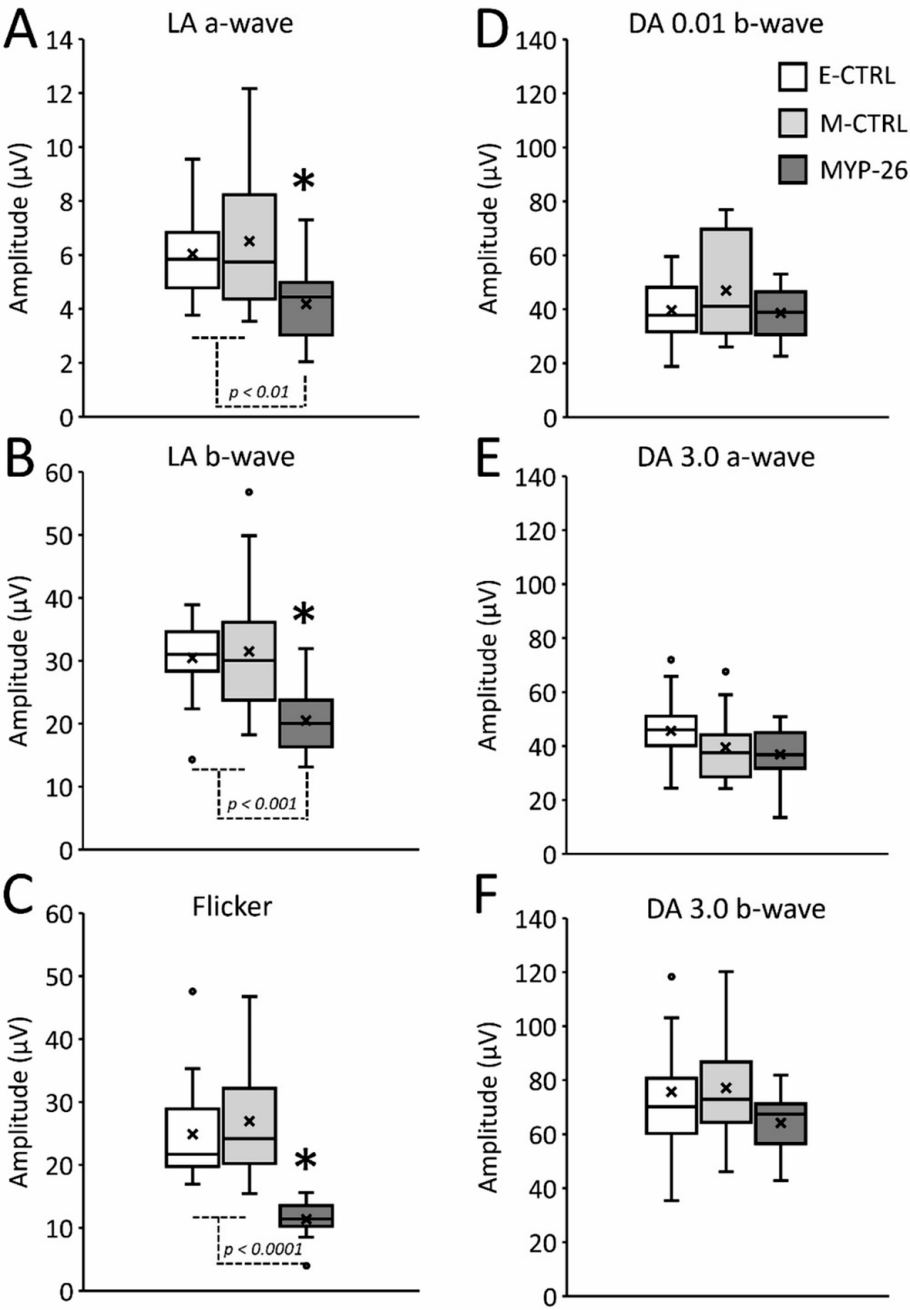
LA and DA ERG amplitudes. The boxplots show mean (X) and median (central lines) values; box = interquartile range [IQR]; whisker = minimum and maximum values. Amplitudes are given in µV for ERG components: E-CTRL (empty), M-CTRL (light grey) and MYP-26 (dark grey). LA flash (**A**-**B**) and flicker (**C**) amplitudes were significantly reduced in MYP-26 group. DA mean amplitudes were comparable among the three groups for DA 0.01 b-wave (**D**), DA 3.0 a-wave (**E**) and DA 3.0 b-wave (**F**). The indicated p values show the results of F-tests; asterisks mark statistically significant differences

Figure 3 shows light adapted (LA) flash (Fig. 3A) and flicker (Fig. 3B) as well as dark adapted (DA) ERGs to weak (Fig. 3C) and standard flash (Fig. 3D). The oscillatory potentials (OPs, Fig. 3E) were isolated from ERG signals obtained with the standard flashes using a band-pass filter of 75 to 300 Hz. The comparison of the averaged signals among the three groups (Fig. 3), revealed abnormal light-adapted flash and light-adapted flicker ERG waveform in MYP-26 patients while E-CTRL and M-CTRL averaged light-adapted traces were comparable to each other (Fig. 3A and B). In contrast, dark-adapted ERGs averaged responses (Fig. 3C and D) were similar among the three groups. OPs slightly differ among the groups (Fig. 3E). Interestingly, light-adapted flash and flicker ERGs of a male carrier were reduced compared to an age-matched male control (Figure S1). The amplitudes were at the same level as the mean amplitudes found in MYP-26 female patients (see Table S2 for the female values and Table S3 for the male values). ERG amplitudes extracted from the above-described components are shown in Fig. 4 LA a-wave (Fig. 4A) and b-wave (Fig. 4B) amplitudes were statistically different among the groups (F_(2, 45)_ = 5.942, *p* = 0.0051 and F_(2, 45)_ = 9.723, *p* < 0.001, respectively). Post-hoc Bonferroni correction revealed that the differences were restricted to the MYP-26 group. LA ERGs were significantly reduced in MYP-26 patients compared to both E-CTRL (*p* = 0.023 and *p* = 0.001 for a- and b-wave, respectively) and M-CTRL (*p* = 0.007 and *p* = 0.001 for a- and b-wave, respectively) groups while E-CTRL and M-CTRL groups showed comparable amplitudes (*p* = 0.999 for both a- and b-wave). Similarly, light-adapted flicker amplitudes (Fig. 4C) were significantly different among the groups (F_(2, 45)_ = 20.200, *p* < 0.0001) with comparable amplitudes between E-CTRL and M-CTRL (*p* = 0.999) and even more severely reduced amplitudes in the MYP-26 group compared to both CTRL groups (*p* < 0.0001). LA changes were restricted to the amplitudes. Peak times were comparable among the groups for the three LA components: a-wave (F_(2, 45)_ = 2.107, *p* = 0.133), b-wave (F_(2, 45)_ = 1.225, *p* = 0.303) and flicker (F_(2, 45)_ = 0.274, *p* = 0.762).

**Fig. 5:**
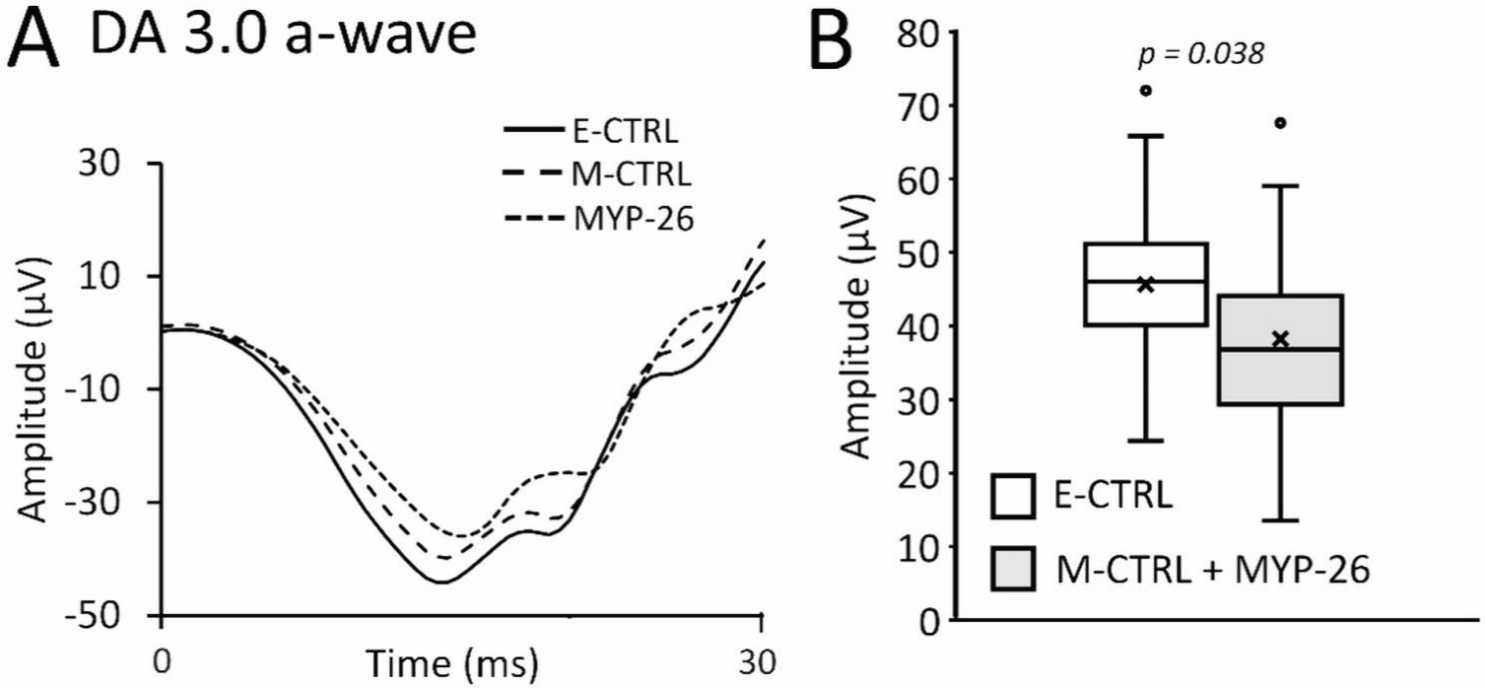
Comparisons of the DA ERG amplitudes. Mean DA 3.0 ERG averaged responses for each group of patients (**A**). The boxplots (**B**) show mean (X) and median (central lines) values; box = interquartile range [IQR]; whisker = minimum and maximum values of a-wave amplitudes (in µV) of the DA 3.0 cd.s/m² ERG. This further analysis revealed slight, but significant, reduced a-wave amplitudes in myopic patients (grey), including patients with and without *ARR3* gene mutation, compared to controls (white)

No effect of *ARR3* gene mutation was observed in DA ERG. However, the presence of myopia itself was associated with slight a-wave reduction (Fig. 4E). DA weak (0.01 cd.s/m²) (Fig. 4D) and standard (3.0 cd.s/m²)(Fig. 4F) b-waves were comparable among the groups (weak flash: F_(2, 45)_ = 1.725, *p* = 0.190 and standard flash: F_(2, 45)_ = 1.511, *p* = 0.232). Similarly, no alterations in b-wave amplitudes were observed when pooling data from high myopic patients (M-CTRL and MYP-26) to be compared with E-CTRL (weak flash: F_(1, 46)_ = 0.634, *p* = 0.430 and standard flash: F_(1, 46)_ = 0.583, *p* = 0.449). In contrast, myopia in general slightly, but significantly (F_(1, 46)_ = 4.543, *p* = 0.038), affected DA 3.0 a-wave (Fig. 5A) when comparing E-CTRL with M-CTRL + MYP-26 (Fig. 5B).

DA OP amplitudes were statistically comparable among the groups either comparing the sum of the OP amplitudes (F_(2, 45)_ = 0.657, *p* = 0.523) or comparing individual DA OP amplitudes (OP2: F_(2, 45)_ = 1.456, *p* = 0.243; OP3: F_(2, 45)_ = 0.915, *p* = 0.407 and OP4: F_(2, 45)_ = 0.674, *p* = 0.515). Similarly, no differences in peak time were observed (OP2: F_(2, 45)_ = 0.011, *p* = 0.989; OP3: F_(2, 45)_ = 0.273, *p* = 0.762 and OP4: F_(2, 45)_ = 0.175, *p* = 0.840). Means and standard deviations (SD) as well as p-values of OP amplitude and peak time comparison can be found in the supplemental material (Table S2).

**Fig. 6:**
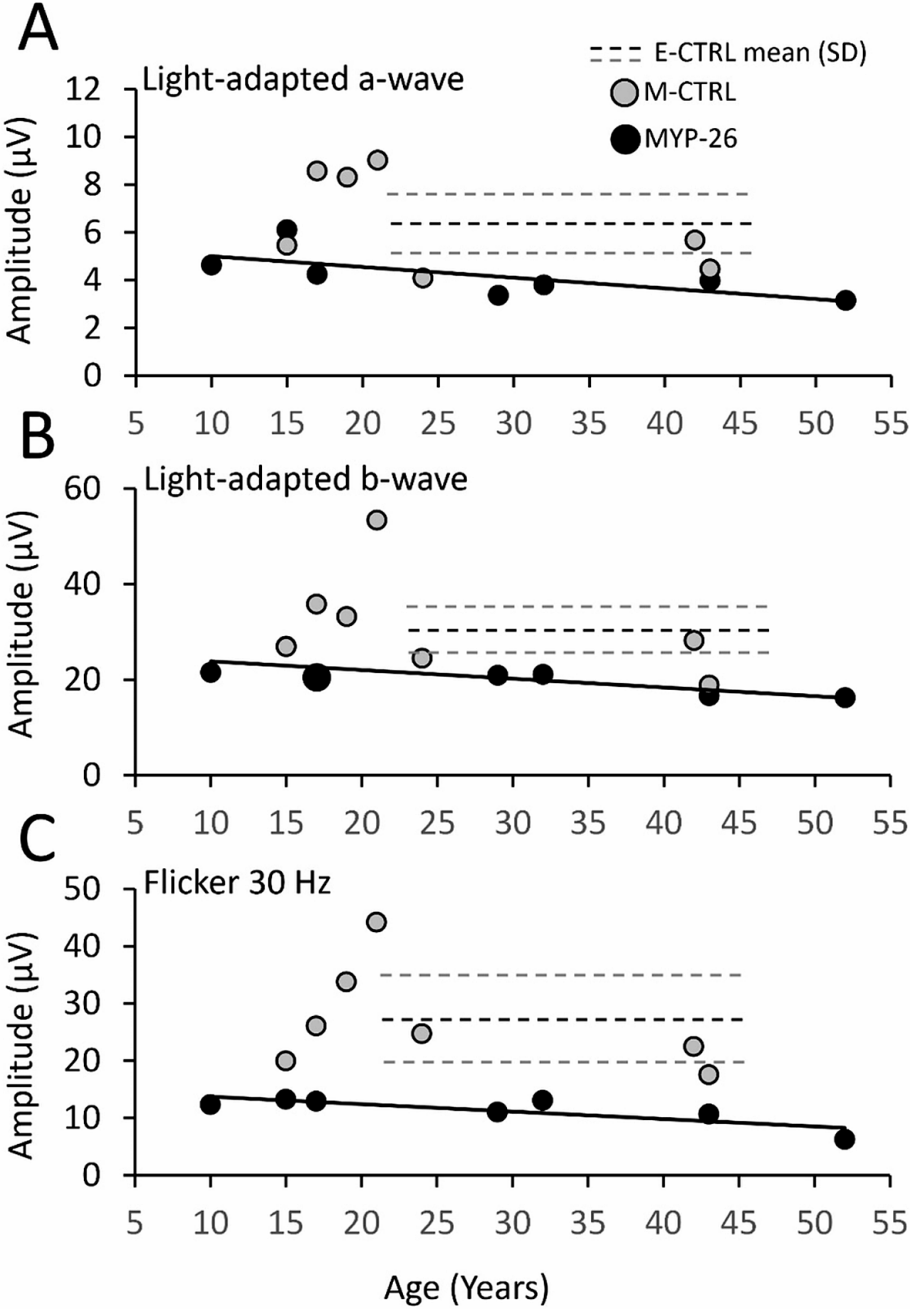
Individual amplitude values of LA components. The values of the LA a-wave (**A**), LA b-wave (**B**) and flicker (**C**) amplitudes are shown as a function of patient’s age for M-CTRL (grey) and MYP-26 (black). Larger symbol represents two data points overlapping. Black dashed lines show control (E-CTRL) means ± SD (grey dashed lines) amplitudes. The mean age of the control group was 31.5 ± 8.8 years (range: 22–47 years). The trendlines (continuous black) were calculated considering only MYP-26 (black) data points

The associations between ERG amplitudes and age are shown in Fig. 6. In MYP-26 patients, individual LA ERG amplitudes slightly decrease with aging. Accordingly, statistical analysis showed a slightly significant negative correlation between ERG amplitudes and age for LA b-wave (rho = -0.59; *p* = 0.028) and flicker (rho = -0.54; *p* = 0.046) amplitudes.

## Discussion

Myopia-26 (OMIM: #301010) is currently claimed to be the most frequent form of non-syndromic Mendelian eoHM, transmitted in an exceptional pattern of X-linked female-limited inheritance [2, 5–7, 19]. The first human identification of *ARR3*-associated Myopia-26 in three Asian families was reported by Xiao et al. in 2016 [9], whereas the first description and phenotypic characterization of the disease in a large multigenerational family of European ancestry has recently been reported by our group [5]. That study revealed ERG alterations associated with selective cone dysfunctions [5]. Shortly afterwards, the ERG phenotype of 11 Myopia-26 patients was reported as mild cone impairment in Asian patients [6].

Refractive error itself implies retinal dysfunctions associated with the axial elongation during myopia progression causing retinal stretching and thinning, decreased retinal cell - in particular, photoreceptor - density, morphological changes in photoreceptor inner and outer segments as well as remodeling of the regular retinal neuron arrangement [10, 14]. These changes modify signal transmission in the retina and consequently affect ERGs. A myopic control group is accordingly necessary to separate the influence of eye elongation factors from the effects of *ARR3* mutation in Myopia-26.

Therefore, to shed light on the ERG phenotype of the *ARR3* heterozygous genotype, we conducted an unbiased analysis of LA and DA responses comparing results of genetically characterized Myopia-26 patients, a hemizygous carrier male and early onset high-myopes without *ARR3* gene mutations, besides the healthy non-HM controls. In addition, LA flash and flicker ERGs were analyzed separately.

Full-field ERGs in high myopia have been extensively reported. To date, no specific ERG profiles have been established for high myopia, most likely due to the heterogeneous genetic backgrounds of the analyzed subjects. According to previous reports, DA ERG responses are more likely to be affected in high myopia [10] and there is no consensus in the literature regarding specific b-wave [20] or combined a-wave / b-wave amplitude changes [16, 20]. The present results, on one hand, confirmed that high myopia is associated with reduced amplitudes of the DA a-wave, regardless of the presence of *ARR3* gene mutation. On the other hand, Myopia-26 was found to be specifically associated with abnormal light adapted cone responses (a-wave and b-wave) as well as flicker ERG alterations. Our results therefore provided electrophysiological evidence of underlying cone dysfunction in Myopia-26 patients and the carrier male, caused by mutations in the X-chromosomal *ARR3* gene.

The cone-arrestin shows a cell-specific pattern of expression primarily in retinal cone photoreceptors and to a lesser extent in pinealocytes of the pineal gland [21]. The functional characterization of *ARR3*-knockout mice suggested that cone-arrestin plays a role in refractive development influencing visual acuity and contrast sensitivity [17]. It is well established that cone dysfunction causing form deprivation may induce refractive error development [22]. Another noteworthy finding of our cohort is that Myopia-26 is associated with reduced BCVA with no advanced stages of pathological fundus alterations or myopic maculopathy (META-PM C3, 4) that could explain it. Low BCVA in Myopia-26 patients may therefore be directly attributed to the cone dysfunction caused by *ARR3* gene mutations. It has been previously shown [23] and recently confirmed [24] that patients with inherited retinal diseases (IRDs) primarily affecting the cone system, such as cone-rod dystrophy, achromatopsia, blue-cone monochromacy, show higher prevalence of myopia than IRDs primarily affecting rods, such as retinitis pigmentosa. These data also support the association between cone dysfunction and refractive error development [22]. In our cohort, structural alterations in cone photoreceptors and accordingly in the retina characteristic of cone dystrophies, i.e. the disruption or even loss of photoreceptor cell layer, were not observed. Therefore, the alteration in cone function (reduced LA ERG responses) described here is better referred to as a cone dysfunction rather than a cone dystrophy.

Two limitations of our study are the conditions that all subjects harboring an *ARR3* mutation are from the same family, and that only a single carrier male could be recruited. The fact that the non-myopic carrier male also displays the ERG alterations can be explained at least two ways: (i) the cone dysfunction is not the cause of myopia development, (ii) besides cone dysfunction, an additional condition, only present in females is necessary for axial elongation. The first explanation is unlikely in light of the literature data discussed above, but the recently observed malfunction of the melanopsin system specific to symptomatic Myopia-26 patients leaves this possibility open [25]. A potential pathomechanism fitting the second hypothesis, also interpretable as a male-protective effect, is described below.

While the first version of this manuscript was under evaluation, a Chinese group reported the results of a ten-year follow-up of *ARR3* heterozygous patients [26]. They reported the progressive worsening of color vision and cone function with age, which agrees with our present findings. Importantly, they found cone dysfunction to dominate in L/M cones. This supports our earlier hypothesis stating that the lack of the cone-arrestin affects L/M cones (without affecting S-cones), potentially leading to their delayed shut-off. The resulting intensification of red sensation may lead to hyperopic defocus, which provides a constant signal for bulbar elongation. The authors introduce yet a further hypothesis, claiming that the heterogeneous nature of the female retina, caused by random X inactivation leads to a signal of “constitutive contrast”, which has also been shown to promote axial elongation [27, 28]. Briefly, this term refers to the retinal signal resulting from heterogeneous L/M cone excitation, which generates axial elongation due to its potential similarity to the signal produced by defocus. This could be viewed as if *ARR3*-linked cone dysfunction only led to myopia if an additional condition, the functional heterogeneity of L/M cones was also granted, explaining why such mutations cause myopia in heterozygous females, but not in hemizygous males.

## Conclusion

Our electrophysiological investigation of emmetropic and myopic subjects revealed dark-adapted ERG a-wave alterations caused by myopic refractive error, which is consistent with previous reports. More importantly, the present data demonstrated that harboring a nonsense allele of *ARR3* leads to a significant cone dysfunction. Such cone dysfunction may play a role in the myopic refractive error development of Myopia-26 patients, but only if a yet unconfirmed condition, only provided in females is granted. Future investigations should consider that the genetic background may strongly influence the ERGs of high myopic patients. In addition, it remains to be explored whether *ARR3*-related cone dysfunction is a progressive or stationary condition by extending the time frame over which phenotypes are monitored.

## Electronic supplementary material

Below is the link to the electronic supplementary material.

Supplementary Material 1

Supplementary Material 2

## Data Availability

The sequencing data used and analysed during the current study are available from the corresponding author on reasonable request. All other data generated or analysed during this study are included in this published article and its supplementary information files.

## Abbreviations

BCVA: best corrected visual acuity
DA: dark-adapted
E-CTRL: emmetropic control group
eoHM: early onset high myopia
ERG: electroretinography
ISCEV: International Society for Clinical Electrophysiology of Vision
LA: light-adapted
M-CTRL: myopic control group
MYP-26: Myopia-26 patient group
OCT: optical coherence tomography
OP: oscillatory potential
PCR: polymerase chain reaction
SE: spherical equivalent
WES: whole exome sequencing
