# Supplementary material for: Cone dysfunction in ARR3-mutation-associated early-onset high myopia: an electrophysiological study
Source: Orphanet J Rare Dis. 2024 Oct 17;19:385. doi: 10.1186/s13023-024-03390-9 (PMC11488070; doi:10.1186/s13023-024-03390-9)
Supplement: Supplementary file 1 — Supplementary Material 1 [file 13023_2024_3390_MOESM1_ESM.docx]

***Supplementary material***

**Cone dysfunction in *ARR3*-mutation-associated early-onset high myopia: an electrophysiological study**

**Table S1. Primer names and the exons of *ARR3* amplified by PCR**

| **Primer name** | **Sequence** | **Spanned exons** |
| --- | --- | --- |
| Arr3_ex2Fw | TGGTATGCTGGAGTGGGGTG | 2, 3, 4, 5 |
| Arr3_ex2Rev | ACTCATCGGGTTACCTGTCTG | 2, 3, 4, 5 |
| Arr3_ex6Fw | ACCACCAAGCCAGGCTAATAC | 6, 7 |
| Arr3_ex6Rev | CCCACTGTTCCTCTTCCCTG | 6, 7 |
| Arr3_ex8Fw | TCTTTGCCCTTGTCCCTTTACA | 8, 9 |
| Arr3_ex8Rev | GCCTGCCCAAGTAGGAAATC | 8, 9 |
| Arr3_ex10Fw | GGCAATAGCGCTAAGGAAGC | 10, 11 |
| Arr3_ex10Rev | GATGGGACTCCTGGGGGTAA | 10, 11 |
| Arr3_ex12Fw | CCCACCAGAACTAATTTCTCCC | 12 |
| Arr3_ex12Rev2 | TGGTCCCACAGGCATTATTGAG | 12 |
| Arr3_ex13Fw | AGGGTGTGGTGATAGGAATGC | 13, 14 |
| Arr3_ex13Rev | CCCTCAGACCCTCCAGAATCA | 13, 14 |
| Arr3_ex15Fw | AACTAAGGGAGGGAGGTTCAG | 15, 16 |
| Arr3_ex15Rev | CACCCCAAGGAAGCATTTTCA | 15, 16 |
| Arr3_ex17Fw | AAAAAGAGTGGACACGGAGGA | 17 |
| Arr3_ex17Rev2 | CCAGGAAGAGGAAATAGGCAAGAG | 17 |

**Table S2. ERG results (mean ± standard deviation)**

**Table S3. ERG values of male *ARR3* carrier compared to an age-matched male control.** Mean ± standard deviation of both eyes for the control and individual values from each eye for the male carrier patient.

*amblyopic eye

**ERG results**

*Description of the ERG waveforms based on Figure 1 of the manuscript*

LA flash responses displayed an initial negative component (a-wave) in which the peak amplitude was at about 12 ms after the flash onset followed by a larger positive component (b-wave) at about 28 ms peak amplitude. LA ERG a-wave and b-wave are known to originate in cones / hyperpolarizing-bipolar cells and cone-to-bipolar cell synapses, respectively ^1,2^. LA flicker ERG displayed 30 Hz sine-like responses representing cone-driven activation of cone-to-bipolar cell synapses ^3^. DA ERG to weak flashes elicited a rod-dominated response with only a positive component (b-wave) peaking at about 85 ms. This component represents the activation of rod-to-On (depolarizing) bipolar cell synapses. The DA ERG to standard flash elicited a response containing a-wave, at around 15 ms, and b-wave, at around 48 ms. The a-wave represents photoreceptors’ and bipolar cells’ activation and the b-wave is mainly originated at the On-bipolar cells ^2,4^. The oscillatory potentials (OPs) were isolated from ERG signals obtained with the standard flashes using a band-pass filter of 75 to 300 Hz. They are thought to originate at the amacrine cells through feedback interactions taking place in the inner retina ^5^. **Table S2** shows mean ± standard deviation of ERG results.

*Light-adapted and dark-adapted peak times*

Peak times were comparable among the groups for the three LA components: a-wave (F_(2,47)_ = 2.107, p = 0.133), b-wave (F_(2,47)_ = 1.225, p = 0.303) and flicker (F_(2,47)_ = 0.274, p = 0.762). They were also comparable among the groups for the three DA components: b-wave weak flash (F_(2,47)_ = 0.423, p = 0.657), a-wave standard flash (F_(2,47)_ = 0.331, p = 0.720) and b-wave standard flash (F_(2,47)_ = 0.884, p = 0.420).

*Dark-adapted oscillatory potentials*

DA OP amplitudes were statistically comparable among the groups either comparing the sum of the OP amplitudes (F_(2, 47)_ = 0.657, p = 0.523) or comparing individual DA OP amplitudes (OP2: F_(2, 47)_ = 1,456, p = 0.243; OP3: F_(2, 47)_ = 0.915, p = 0.407 and OP4: F_(2, 47)_ = 0.674, p = 0.515). Similarly, no differences in peak time were observed (OP2: F_(2, 47)_ = 0.011, p = 0.989; OP3: F_(2, 47)_ = 0.273, p = 0.762 and OP4: F_(2, 47)_ = 0.175, p = 0.840). Means and standard deviations as well as p-values of OP amplitude and peak time comparison can be found in supplemental material (**Table S2**).

*ERGs of male carrier patient*

In addition to full-field ERGs recorded from the female groups, two male subjects were examined: one male control (37 years old, spherical equivalent = -2.50 D and BCVA = 1.0 / 1.0 in both eyes) and one *ARR3* carrier male (37 years old, spherical equivalent = -1.00 / -2.00 D, BCVA = 1.0 / 0.7, left eye amblyopic due to congenital exotropia). The examinations followed the same procedures and used identical equipment and ISCEV standard protocols as the ones used and applied in the female groups.

Light-adapted (LA) ERG responses from an age/refraction-matched male control subject and the *ARR3* carrier male are shown in **Figure S1** for both, flash (A) and flicker (B) ERG responses. LA ERG responses were reduced in both eyes and in both protocols for the male carrier compared to the male control. No remarkable differences in DA ERGs were observed (data not shown).

**Table S3** shows that LA ERG a-waves and b-waves were reduced in the male carrier by about 30% of the control values. Flicker ERG amplitudes were more serverely reduced (~10 µV), to about 50% of the the control mean value (20.5 µV). Implicit times of the flash ERG a-wave and b-wave and of the flicker ERG were comparable between the control and the male carrier.

**Figure S1. ERGs from a male *ARR3* carrier compared to an age-matched male control.** Light-adapted 3.0 cd.s/m² (A) and light-adapted flicker (B) ERG traces recorded from an age-matched male control (mean of the eyes) and the right and the left eyes, respectively, from a the male carrier with confirmed mutation in the *ARR3* gene. RE = right eye and LE = left eye.

**
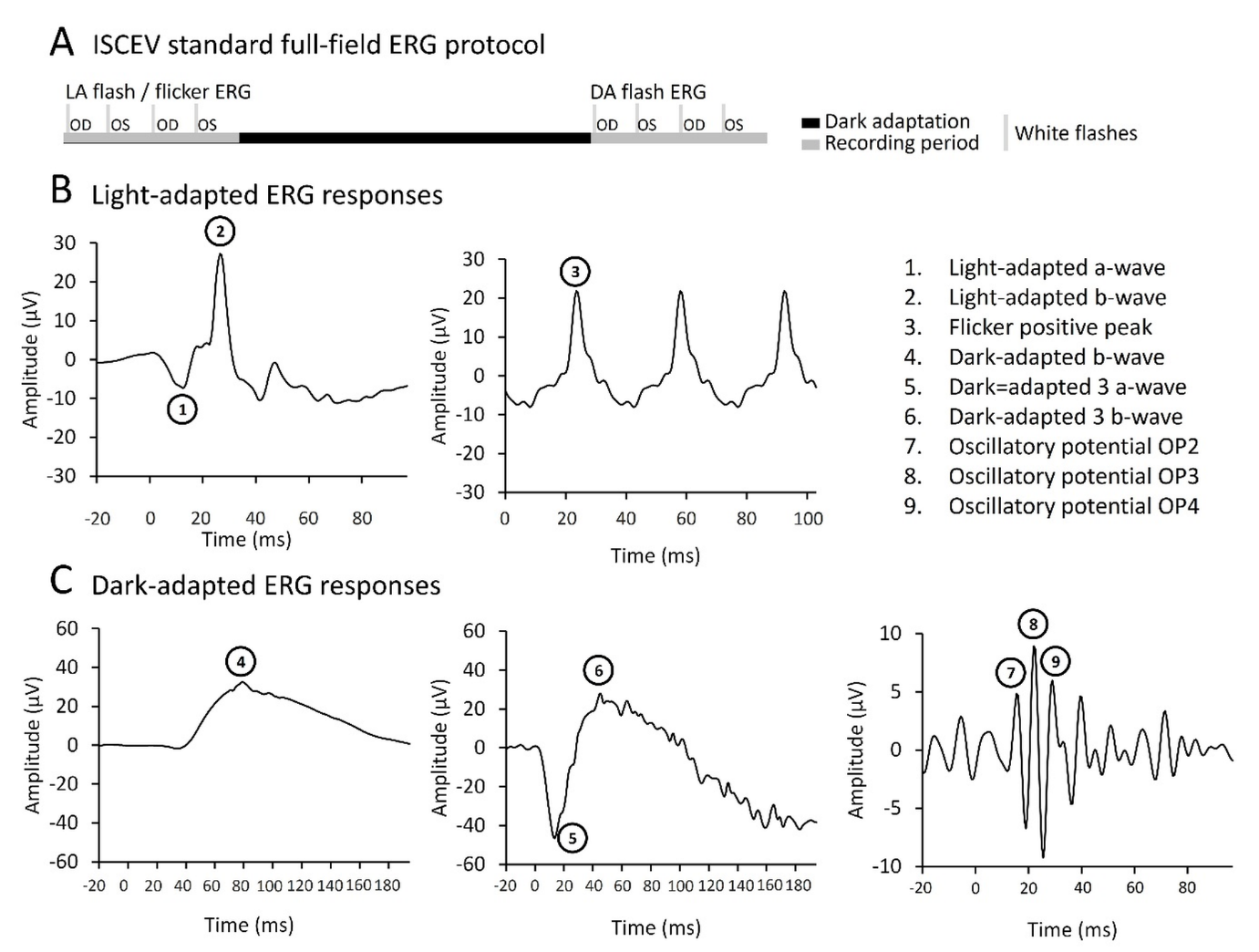
**

**Figure S2. ISCEV Standard Full-field ERG protocol and components.** The protocol consisted of the light-adapted (flash and flicker) ERG recordings followed by the dark-adapted (weak and standard flash) ERG measurements with a 10-minute interval of dark-adaptation (A). Light adapted (B) and dark-adapted (C) ERG responses were analysed as follows: amplitude of the negative peaks (light-adapted flash and dark-adapted 3 a-waves, numbers 1 and 5) was the difference in μV between the baseline and the minimum amplitude value at the first trough after stimulus onset. The amplitude of the positive peaks (b-waves and flicker / OP peaks, numbers 2, 3, 4, 6 and 7-9) was the difference in μV between baseline or a-wave trough and the peak amplitude at the positive component. The implicit times in milliseconds corresponded to the intervals between the stimulus onset and the peak amplitudes.

**REFERENCES**

1. Bush, R. A. & Sieving, P. A. A proximal retinal component in the primate photopic ERG a-wave. *Invest. Ophthalmol. Vis. Sci.* **35**, 635–645 (1994).

2. Frishman, L. J. Origins of the electroretinogram. in *Principles and practice of clinical electrophysiology of vision* 139–183 (MIT Press, Cambridge, 2006).

3. Robson, A. G. *et al.* ISCEV Standard for full-field clinical electroretinography (2022 update). *Doc. Ophthalmol. Adv. Ophthalmol.* **144**, 165–177 (2022).

4. Robson, J. G. & Frishman, L. J. The rod-driven a-wave of the dark-adapted mammalian electroretinogram. *Prog. Retin. Eye Res.* **0**, 1–22 (2014).

5. Wachtmeister, L. & Dowling, J. E. The oscillatory potentials of the mudpuppy retina. *Invest. Ophthalmol. Vis. Sci.* **17**, 1176–1188 (1978).
