# Supplementary material for: Cone dysfunction in ARR3-mutation-associated early-onset high myopia: an electrophysiological study
Source: Orphanet J Rare Dis. 2024 Oct 17;19:385. doi: 10.1186/s13023-024-03390-9 (PMC11488070; doi:10.1186/s13023-024-03390-9)
Supplement: Supplementary file 2 — Supplementary Material 2 [file 13023_2024_3390_MOESM2_ESM.docx]

Response to the Reviewer report:

*Thank you for this revised manuscript.A number of small changes still need attention.*

Response: We cordially thank the reviewer for the record amount of time dedicated to our manuscript.

*IN the abstract,  in results the authors on several occasions use the abbreviation "cca", eg, "the prior values were reduced by cca. 35% ". This is not used in the manuscript, What is this? what does it mean.*

Response: The abbreviation “cca.” refers to the latin word circa, meaning “approximately. To avoid misunderstandings, we replaced both instances of “cca.” with tildes (~), which are widely used typographic symbols resembling “approximately”. (We also attached a tracked version of the manuscript for your convenience.)

*Similarly in the abstract, and in the manuscript the abbreviation "F(2,47)" and also F(1,47) is used multiple times. Please explain this term*

Response: In brief, we used analyses of variance (ANOVAs) to screen for differences among groups. The ANOVA uses the F-test statistic, which is the following ratio: the population variance estimated from group means / the population variance estimated as average of group variances. To test whether this ratio (the F value) is higher than that expected from random variation (in other words, provide a p value), one must take into account the degrees of freedom (df) of the numerator, and the degrees of freedom of the denominator. The prior is the number of groups m minus one (m-1), and the latter is the number of groups m times the size n of each group minus one [m(n-1)]. These two df values are indicated in parentheses after the letter F to denote the mode of calculation of the p value from the F-ratio [e.g. F(2,47)]. Since this is textbook information, we decided not to include this in our publication.

We thank the reviewer for bringing up this issue, for we discovered an incorrect df in the previous version of the manuscript: the second number erroneously displayed the total df, and not the df of the denominator. We corrected the display, and double checked the F-test output. Only minor changes were found, and only in a few cases (also available tracked).

*p8, line 54 "hat" is presumably "that"*

Response: The correction has been made.

*p 9 discusses the one male patient in the study who had reduced LA flash and flicker compared with male controls. But there were no male controls in this cohort - this needs to be clarified what control data was used.*

Response: We thank the reviewer for calling our attention to updating the text. We had a male control indeed, which we described in the Additional File, but not in the main text of the previous version.

We updated the Abstract:

“Participants were 26 volunteers: 10 healthy control females (E-CTRL, mean age=31.5±8.8 years), one healthy control male, one carrier male of the mutant *ARR3* allele and 14 female eoHM patients (mean age=27.0±13.1 years) divided in two groups: seven without (M-CTRL) and seven with (MYP-26) genetic alteration in the *ARR3* gene.”

We also modified the Participants section of the Methods:

“One male carrier, hemizygous for the *ARR3* mutation was also included, along with a male emmetropic control.”

We thank the critical comments again, and hope that this time our manuscript will meet all expectations.
